# Retrospective Analysis of a WashU-Based Knowledge-Based Planning (KBP) Model for Breast and Chest Wall Planning in Guatemala: Compliance With American Society for Radiation Oncology (ASTRO) 2026 Guidelines

**DOI:** 10.7759/cureus.110611

**Published:** 2026-06-10

**Authors:** Milton E Ixquiac Cabrera, Erick O Montenegro, Matthew Schmidt, Baozhou Sun, James A Kavanaugh, Taoran Li, Angel Velarde, Vicky de Falla, Francisco Reynoso

**Affiliations:** 1 School of Physical and Mathematical Sciences, Universidad de San Carlos de Guatemala, Guatemala, GTM; 2 Radiotherapy, Liga Nacional Contra el Cáncer, Guatemala, GTM; 3 Radiation Oncology, Washington University School of Medicine, St. Louis, USA; 4 Medical Physics, Baylor College of Medicine, San Antonio, USA; 5 Radiation Oncology, Mayo Clinic, Rochester, USA; 6 Department of Radiation Oncology, University of Pennsylvania, Philadelphia, USA; 7 Oncology, Oncological Hospital Incan, Guatemala, GTM; 8 Medical Science Liaison, Varian Medical Systems, Schiller Park, USA

**Keywords:** astro guidelines, breast cancer, chest wall, dosimetric quality, hypofractionation, knowledge-based planning, low- and middle-income countries, rapidplan, vmat

## Abstract

Background/purpose

Breast cancer is among the most prevalent malignancies treated at the Liga Nacional Contra el Cáncer (LNCC) in Guatemala, representing a significant proportion of annual radiotherapy cases. Access to high-quality, standardized treatment planning in resource-constrained settings remains a critical challenge. This study evaluates the dosimetric performance of knowledge-based planning (KBP) models adapted from Washington University (WashU) in St. Louis for breast and chest wall radiotherapy at LNCC, validated against a retrospective 2025 clinical cohort, and benchmarked against the ASTRO 2026 Practical Radiation Oncology guidelines.

Materials and methods

A retrospective analysis of 84 treatment plans (40 left, 44 right) for whole-breast or chest-wall treatment with regional nodal involvement was performed. All patients were treated using volumetric modulated arc therapy (VMAT) under a moderate hypofractionation scheme (40.05 Gy in 15 fractions). KBP models were developed during 2022-2024 using Eclipse V18.0 (Varian Medical Systems, Palo Alto, CA) RapidPlan^TM^, originally built at Washington University, and augmented with 194 left-breast and 103 right-breast cases from LNCC. Model validity was confirmed with Varian’s model analytics tool. Dosimetric metrics for the planning target volume (PTV) and organs at risk (OARs) were extracted using a custom ESAPI (the OWASP enterprise security application programming interface) application and compared against ASTRO 2026 Table 3 benchmarks, categorized as recommended (green), acceptable (yellow), or unacceptable (red).

Results

PTV coverage was adequate, with an average V95% of 97.3% ± 1.8%; V90% of 99.7% ± 0.4 (right), and average V95% of 96.6%±3.8%; V90% of 99.0%, ± 2.8% (left).

Most plans met ASTRO's recommended range. The heart mean dose was well-controlled, with median values of 2.4 Gy (right) and 4.2 Gy (left). Ipsilateral lung V18Gy showed a median of 18.6% (right) and 17.7% (left), and V10Gy of 33.3% (right) and 31.6% (left), both within acceptable ranges. Spinal cord D0.035cc had a median of 8.3 Gy (right) and 10.1 Gy (left), well below neurological tolerance thresholds. Contralateral breast D10% had median doses of 2.6 Gy (right) and 2.9 Gy (left), with ranges of 1.7-3.1 Gy and 2.2-5.4 Gy, respectively. KBP model analytics validation confirmed institutional model statistics fell within acceptable quality benchmarks across all evaluated structures.

Conclusion

KBP models developed at a high-income institution and iteratively refined with local data can be successfully deployed for breast and chest wall radiotherapy in a resource-constrained low- and middle-income country (LMIC) setting, achieving dosimetric outcomes consistent with ASTRO 2026 guidelines. The temporal separation between model training (2022-2024) and retrospective validation (2025) further confirms the models’ robustness and generalizability. This approach supports standardized, high-quality treatment planning at scale, contributing to more equitable access to radiotherapy for breast cancer patients.

## Introduction

In Latin America, cervical and breast cancer represent a critical public health challenge due to their high incidence and mortality. In Guatemala, specifically at the National League Against Cancer (*Liga Nacional Contra el Cáncer* or LNCC), these pathologies rank first and second in frequency, respectively, underscoring the need to implement cutting-edge treatments that optimize clinical outcomes [1,2].

In recent years, LNCC has undergone a significant technological transformation through the adoption of new equipment and treatment techniques. In breast cancer treatment, moderate hypofractionation has been implemented following evidence from the UK START-B trial, which established the non-inferiority of 40.05 Gy in 15 fractions over 3 weeks compared with the conventional 50 Gy in 25-fraction standard, with equivalent local tumor control and late normal tissue effects [3]. This approach reduces overall treatment duration while maintaining radiobiological efficacy and has subsequently been adopted as the standard of care in many international centers and adapted for nodal irradiation settings.

A fundamental pillar of this advancement has been the collaboration with Washington University (WashU) in St. Louis, initiated in 2018. This quality transfer project has facilitated technical support and knowledge exchange for the creation of RapidPlan models (Varian Medical Systems, Palo Alto, CA). These models, based on artificial intelligence for dose prediction, have been adapted to the specific case mix of the Guatemalan patient population, enabling more efficient, standardized planning.

The present study aims to: (1) audit institutional plan quality by retrospectively evaluating the dosimetric performance of KBP-generated volumetric modulated arc therapy (VMAT) plans against ASTRO 2026 guideline-based constraints [4]; (2) validate the robustness of the locally adapted KBP models using a patient cohort temporally independent from the training dataset; and (3) demonstrate the feasibility of inter-institutional KBP model transfer to a resource-constrained low- and middle-income country (LMIC) clinical setting. This is a retrospective dosimetric study; clinical outcomes, toxicity, and health-equity endpoints were not measured. VMAT was selected for breast or chest wall irradiation with locoregional lymph nodes due to its delivery efficiency and ability to accommodate large patient volumes while maintaining plan quality. Shorter treatment times and fewer beam arrangements improve throughput, setup reproducibility, and patient tolerance, particularly in a high‑volume setting like LNCC.

VMAT is well-suited to the large, geometrically complex target volumes encountered when axillary, supraclavicular, and internal mammary nodes are included. Conventional 2D or 3D conformal techniques often cannot achieve adequate target coverage in this setting without exceeding heart, lung, or other organ‑at‑risk dose constraints. Although inverse planning improves conformity, low‑dose metrics, such as mean heart dose, can be challenging to control due to increased monitor units, small effective aperture sizes, and greater modulation complexity, which can contribute to broader low‑dose spread. Careful VMAT design using partial arcs and avoidance sectors mitigates these effects while maintaining conformal coverage and improving dose homogeneity. Improved homogeneity reduces hotspots and dose variability, which are associated with better cosmetic outcomes and reduced fibrosis risk, particularly in breast-conservation and reconstructed-chest-wall patients. Overall, VMAT provides an efficient and clinically appropriate technique when simpler approaches cannot reliably meet coverage, organ‑at‑risk, and homogeneity objectives.

## Materials and methods

A retrospective dosimetric analysis of 84 treatment plans for breast cancer (40 left and 44 right) was performed, including both whole breast and chest wall patients with regional lymph node involvement. All patients were treated under a moderate hypofractionation scheme (40.05 Gy in 15 fractions), and dosimetric outcomes were benchmarked against ASTRO 2026 guidelines [4]. Of the 84 patients included, approximately 70% underwent post-mastectomy chest wall irradiation, with the remainder receiving whole-breast treatment. All patients received regional nodal irradiation encompassing the supraclavicular fossa and axillary levels. Internal mammary chain coverage was not routinely included in this cohort. Reconstruction status was not systematically recorded in the dosimetric dataset.

Planning system and treatment technique

Planning was carried out in the Eclipse™ treatment planning system (TPS) version 18.0 (Varian Medical Systems). The VMAT technique was used with a four-segment configuration, optimized for each laterality: left breast - arcs between 179° and 305° (clockwise and counterclockwise); right breast - arcs between 55° and 181° (clockwise and counterclockwise). The optimization process followed strategies for creating virtual skin flash [5] and institutional planning guidelines from Washington University in St. Louis.

**Figure 1:**
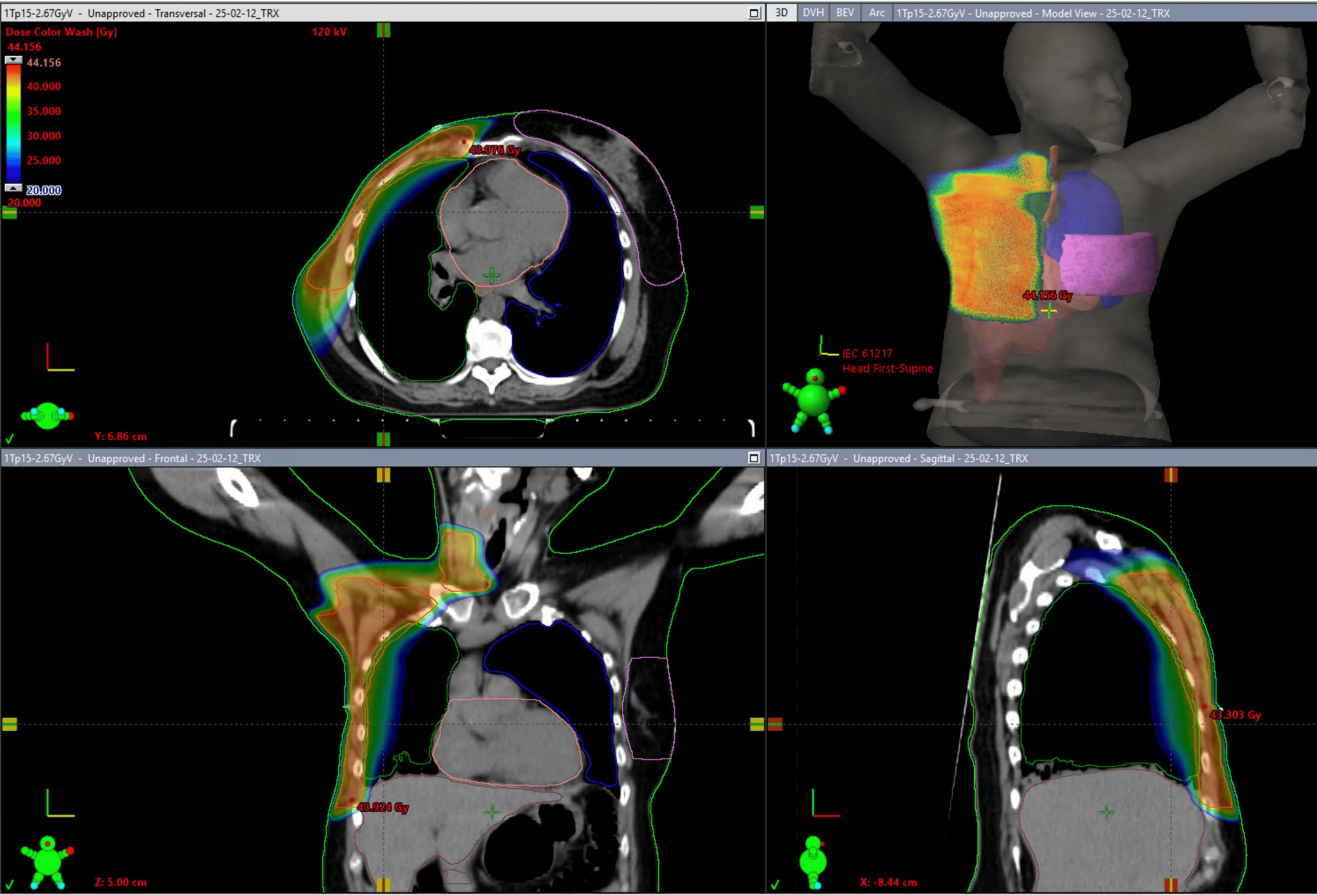
Dose distribution for the right chest wall Shows good conformal coverage with dose falling off rapidly. The lower threshold in the dose distribution is 15 Gy, which typically falls within 2.5 cm of the target edge, sparing the lung, heart, and other medial structures.

Many of our patients received chest wall treatment. In this case, the planning process is a bit more complex due to the short distance between the lung and the out-of-field region, as shown in Figure 1. The skin flash technique covers the respiratory movement, but the volume to be irradiated is small. The planning target volume (PTV) cropped from the body limit is 4 mm. Additionally, we create a new volume called “z_vias”; this volume includes the airways, such as the trachea, esophagus, and larynx, and an additional 3 mm is added to avoid the PTV, with a 4 mm margin.

Implementation of knowledge-based planning (KBP)

For plan optimization, a knowledge-based (KBP) RapidPlan™ model was used, originally developed by Washington University in St. Louis and adapted to the specific case mix of the Guatemalan patient population at LNCC, adding 194 cases to the left breast model and 103 cases to the right breast model. Both models were validated using Varian's model analytics tool, which evaluates the statistical integrity of the training dataset by analyzing dose-volume histogram regression coefficients and identifying cases with significant geometric or dosimetric deviations. This validation process was carried out with appropriate institutional oversight, confirming that model statistics remained within acceptable quality benchmarks across all evaluated structures. These models integrate the capability to optimize plans for both the mammary gland and the chest wall with nodal coverage [6]. The optimization constraints used in both models are summarized in Table 1.

**Table 1:** Left and right models' structures and objectives PTV: planning target volume; gEUD: generalized equivalent uniform dose

Structure ID	Constraint	Volume (%)		Dose (%)	Priority	gEUD a	Volume (%)	Dose (%)	Priority	gEUD a
		Left Breast/Chest Wall					Right Breast/Chest Wall			
PTV_40.05Gy	Upper	0	102		130	-	0	102	130	-
	Lower	100	100		130	-	100	100	130	-
Z_PTV_A	Upper	0	102		130	-	0	102	130	-
	Lower	100	100		130	-	100	100	130	-
Z_PTV_EXP	Upper	0	102		Generated	-	0	102	Generated	-
	Lower	100	100		75	-	100	100	75	-
Breast_R/L (contralateral)	Upper	0	5		Generated	-	0	5	Generated	-
	Upper	7	2.5		220	-	2	2.5	220	-
	Target gEUD	-	3		Generated	7	-	3.5	Generated	7
	Line (Pref. Target)	Generated	Generated		Generated	-	Generated	Generated	Generated	-
Esophagus	Line (Pref. Target)	Generated	Generated		Generated	-	Generated	Generated	Generated	-
Heart	Upper	5	Generated		Generated	-	5	Generated	Generated	-
	Upper	30	Generated		Generated	-	30	Generated	Generated	-
	Mean	-	Generated		Generated	-	-	Generated	Generated	-
	Organ gEUD	-	Generated		Generated	1	-	Generated	Generated	1
	Line (Pref. Target)	Generated	Generated		Generated	-	Generated	Generated	Generated	-
Humerus_L	Upper	0	34.5		115	-	0	34.5	115	-
	Organ gEUD	-	29		115	40	-	29	115	40
Liver	Mean	-	Generated		Generated	-	-	Generated	Generated	-
	Line (Pref. Target)	Generated	Generated		Generated	-	Generated	Generated	Generated	-
	Upper	-	-		-	-	10	10	Generated	-
Lung R/L (ipsilateral)	Upper	15	40		130	-	10	21	Generated	-
	Upper	Generated	Generated		Generated	-	35	5	Generated	-
	Upper	-	Generated		95	-	50	2.5	Generated	-
	Upper	-	Generated		95	-	15	40%	145	-
	Upper	Generated	Generated		95	-	25	9	Generated	-
	Mean	-	45		Generated	-	-	Generated	Generated	-
	Organ gEUD	-	40		Generated	40	-	Generated	Generated	3
	Line (Pref. OAR)	Generated	Generated		Generated	-	Generated	Generated	Generated	-
Lung R/L (contralateral)	Upper	10	8		Generated	-	12	1.7	Generated	-
	Upper	5	Generated		Generated	-	5	2.5	Generated	-
	Organ gEUD	-	Generated		Generated	4	-	Generated	Generated	4
	Line (Pref. OAR)	Generated	Generated		Generated	-	Generated	Generated	Generated	-
SpinalCord	Upper	0	9		Generated	-	Generated	9	Generated	-
	Organ gEUD	-	9		Generated	40	-	9	Generated	37
	Line (Pref. Target)	Generated	Generated		Generated	-	Generated	Generated	Generated	-
Thyroid	Organ gEUD	-	87		Generated	37	-	87	Generated	37
Z_vias	Line	Generated	Generated		Generated	-	Generated	Generated	Generated	-

Data extraction and analysis

The results obtained were statistically compared with the quality standards published by ASTRO in the Practical Radiation Oncology guide (2026) [7]. The dosimetric constraints used for plan evaluation are summarized in Table 2. For data visualization, box plots were used, with results categorized by color according to the degree of compliance: Green -recommended values (Ideal); Yellow - acceptable values (acceptable technical variation); Red - unacceptable values.

**Table 2:** Recommended constraints from the ASTRO guide Recommended constraints for “Breast/chest wall with regional lymph nodes: moderately hypofractionated 15 or 16-fraction regimens (2.66-2.67 Gy per fraction to 40.05-42.56 Gy) [7]”.

Organ/Target	Laterality	Metric	Primary goal	Secondary goal	Deviation
Breast (Contralateral)	Left/right	D10%	≤3 Gy	≤5 Gy	>5 Gy
Heart	Left	Mean	≤3 Gy	≤5 Gy	>5 Gy
	Right	Mean	≤1.6 Gy	≤2.4 Gy	>2.4 Gy
Lung (Contralateral)	Left/right	V4.3Gy	≤15%	-	>15%
Lung (Ipsilateral)	Left/right	V18Gy	≤35%	≤40 %	>40%
	Left/right	V10Gy	≤60%	-	>60%
Spinal Cord	Left/right	D0.035cc	≤37.8 Gy	≤42 Gy	>42 Gy
Breast/Chest Wall	Left/right	Rx dose	V95%	V90% >= 90%	V90%<90%
	Left/right	D0.035cc	≤110%	≤115%	>115%
	Left/right	V107%	≤10cc	-	>10cc
Abbreviations: D=dose, PTV=planning target volume, Rx=prescription; V=volume. Goals do not include dose contribution from a boost (eg, lumpectomy cavity boost).					
Note: For Breast/Chest Wall metrics, field volume may substitute for contoured planning target volume (PTV).					

The collection of dosimetric metrics for organs at risk (OARs) and the PTV was automated using ESAPI extraction, a custom application developed on the Eclipse application programming interface (Eclipse scripting API) [8]. Prior to deployment, the ESAPI extraction tool was verified against manual dose-volume histogram (DVH) extraction for a representative subset of plans to confirm the consistency and accuracy of the reported dosimetric values.

This retrospective study used only fully anonymized data and did not involve any direct clinical intervention or patient interaction. In accordance with the LNCC institutional research governance framework, formal ethics committee review was waived for this retrospective study, as it involved exclusively anonymized data from completed clinical treatments with no direct patient intervention. All procedures were conducted in strict compliance with applicable data protection principles, ensuring the confidentiality and privacy of all patient information throughout the analysis.

## Results

**Figure 2:**
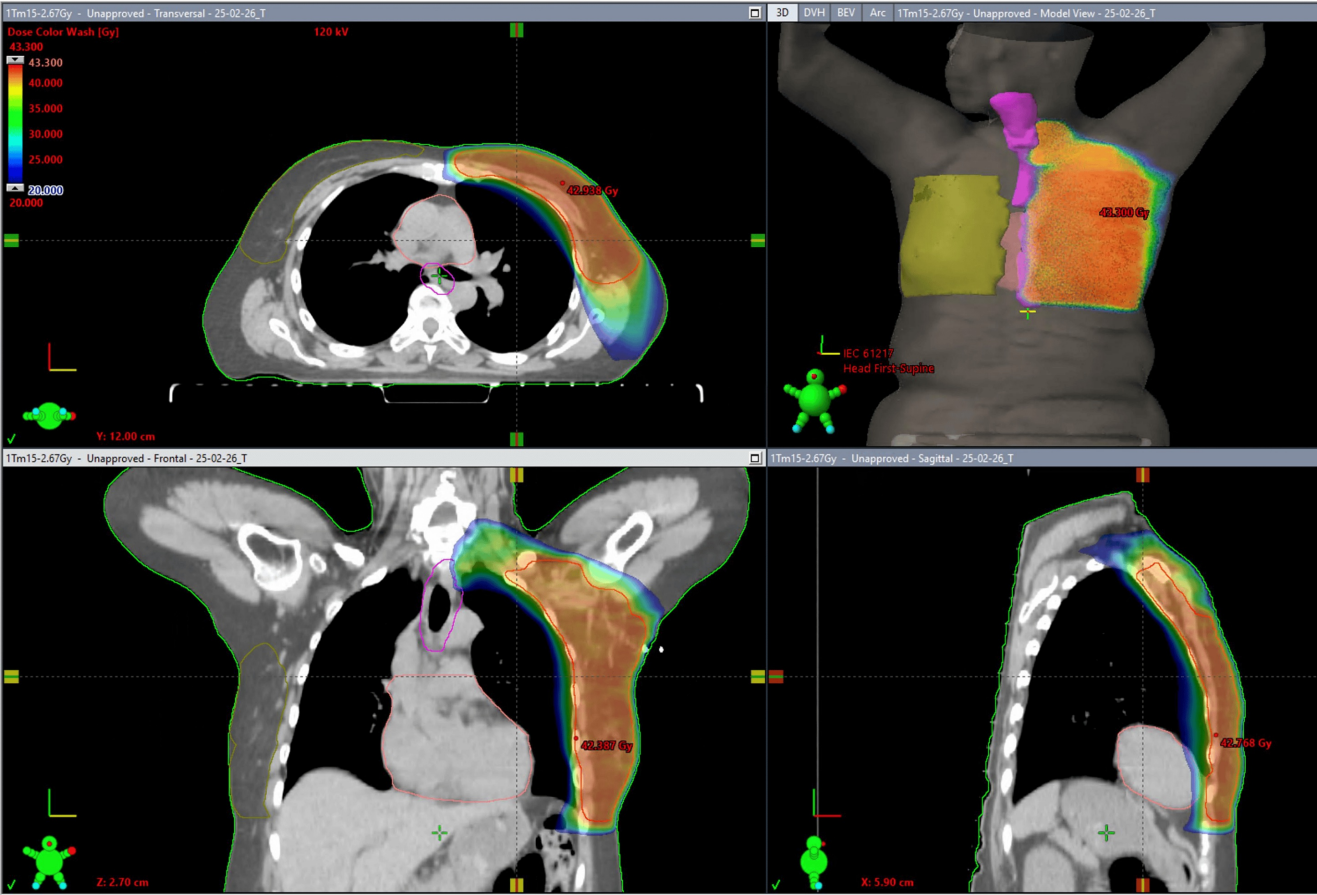
A left breast treated with semiarcs Shows good conformal coverage with a tight dose drop to the organ at risk. The fuchsia-colored volume corresponds to the z_vías, which include organs with air-filled regions, such as the trachea, larynx, and esophagus.

The dosimetric performance and validation of the KBP models, developed and refined between 2022 and 2024, were validated using a cohort of 84 patients treated in early 2025. The results demonstrated high fidelity in target volume coverage, with a median V95% of 97.3%, 1.8% SD; V90% of 99.7%, 0.4 SD (right); and average V95% of 96.6%, 3.8% SD; V90% of 99.0%, 2.8% SD (left). Heart sparing remained optimal, showing a median Dmean of 2.4 Gy (right) and 4.2 Gy (left), while ipsilateral lung V18Gy stayed within acceptable ranges (median 18.6% and 17.7%) (Figure 2).

This temporal separation between model training and clinical validation confirms the model’s robustness and its ability to maintain a high dosimetric standard, consistent with the ASTRO 2026 guidelines, across different patient cohorts.

Protection of OARs

The dosimetric analysis revealed strict dose control in critical organs, consistent with the constraints specified in the Practical Radiation Oncology guide (2026) [7], as summarized in Table 2. Heart: the global mean dose (Dmean) remained at reasonable levels with a median of 2.4 Gy and 4.2 Gy (right and left, range: 1.7-4.5 Gy and 2.2-6.1 Gy, respectively). In left breast cases (n = 40), the VMAT technique kept heart mean doses below the recommended secondary dose limits in the majority of cases. 

**Figure 3:**
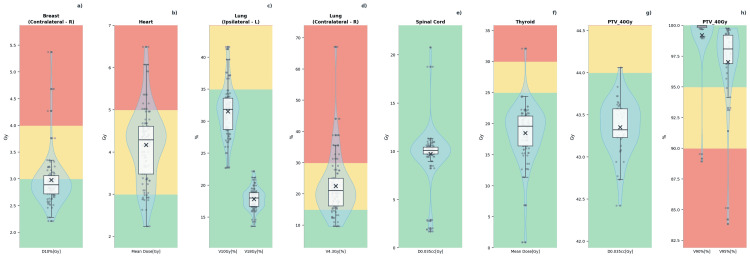
Violin-plot distributions of dosimetric metrics for organs at risk (OARs) and the breast/chest wall planning target volume (PTV) across 40 patients (left side, panels a–h) Each panel presents the median, interquartile range, and full data range for a single dosimetric metric: a) Contralateral breast D10% (Gy); b) Heart mean dose (Gy); c) Ipsilateral lung V10Gy and V18Gy (%); d) Contralateral lung V4.3Gy (%); e) Spinal cord D0.035cc (Gy); f) Thyroid mean dose (Gy); g) Breast/chest wall D0.035cc (Gy); h) Breast/chest wall V90% and V95% (%). Background shading indicates compliance with Table 3 of the ASTRO 2026 Guide: green = recommended (ideal), yellow = acceptable (minor variation), red = unacceptable. Image Credits: Milton Ixquiac (generated with R 4.5.1)

Ipsilateral lung: the V18Gy metric showed median values of 18.56% and 17.66% (right and left, respectively; range: 0.0-29.6% and 14.3-20.9%, respectively), within the acceptable or recommended variation range. The median V10Gy was 33.3% and 31.6% (right and left, respectively), range: 1.7-45.8% and 22.8-39.6%, respectively, demonstrating the efficiency of the KBP model in low-dose spreading (Figure 3).

Spinal cord: the maximum dose (D0.035cc) was 8.3 Gy (right) and 10.1 Gy (left), with ranges of 2.4-10.2 Gy and 1.7-20.8 Gy, respectively, well below the neurological tolerance threshold, ensuring treatment safety.

Contralateral breast: the D10% was 2.6 Gy and 2.9 Gy (right and left, respectively; range: 1.7-3.1 Gy and 2.2-5.4 Gy, respectively), minimizing the risk of secondary neoplasm induction. 

**Figure 4:**
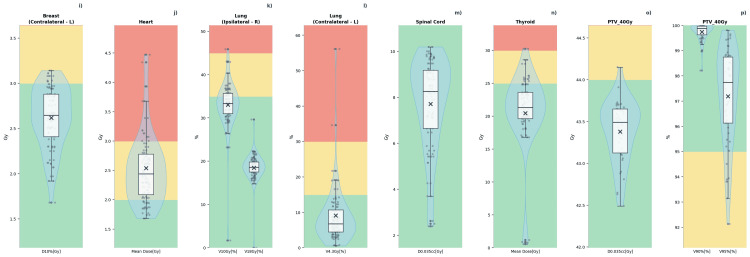
Violin-plot distributions of dosimetric metrics for organs at risk (OARs) and the breast/chest wall planning target volume (PTV) across all 44 patients (44 right side, panels i-p) Each panel presents the median, interquartile range, and full data range for a single dosimetric metric: i) Contralateral breast D10% (Gy); j) Heart mean dose (Gy); k) Ipsilateral lung V10Gy and V18Gy (%); l) Contralateral lung V4.3Gy (%); m) Spinal cord D0.035cc (Gy); n) Thyroid mean dose (Gy); o) Breast/chest wall D0.035cc (Gy); p) Breast/chest wall V90% and V95% (%). Background shading indicates compliance with Table 3 of the ASTRO 2026 Guide: green = recommended (ideal), yellow = acceptable (minor variation), red = unacceptable. Image Credits: Milton Ixquiac (generated with R 4.5.1).

Contralateral lung: V4.3Gy showed median values of 22.9% and 9.1% (left and right, respectively), with ranges of 9.6-67.1% and 0. 6-56.1%, respectively. In the context of VMAT, limiting the low-dose bath to the contralateral lung and the heart is clinically relevant, as even low doses distributed over large volumes have been associated with an increased risk of radiation-induced secondary malignancies through stochastic mechanisms [9,10]. The V4.3Gy threshold in the ASTRO 2026 guidelines reflects this concern, particularly for young patients with long post-treatment life expectancy (Figure 4). These values are consistent with the expected low-dose bath associated with supraclavicular nodal irradiation using VMAT partial arcs, and reflect the inherent trade-off between locoregional target coverage and contralateral lung sparing rather than a model deficiency.

Thyroid: the mean dose was 19.5 Gy (left) and 20.5 Gy (right), with ranges of 6.85-32.1 Gy and 0.6-30.2 Gy, respectively. Minimizing the thyroid dose is an important clinical objective given its proximity to the supraclavicular nodal field: radiation-induced hypothyroidism has been reported in up to 26% of breast cancer patients receiving supraclavicular irradiation, with a mean dose threshold of approximately 21 Gy identified as a significant risk factor [11,12]. Hypothyroidism impairs thyroid hormone production--critical for metabolic regulation, cardiovascular function, and quality of life--and may require lifelong hormonal replacement therapy, underscoring the importance of the thyroid as an OAR in this patient population.

KBP model validation

The adapted RapidPlan models were validated using the model analytics tool (Varian Medical Systems), which evaluates the statistical integrity of the training dataset by analyzing dose-volume histogram regression coefficients and identifying cases with significant geometric or dosimetric deviations. This process enabled a comprehensive audit of the training dataset after incorporating local patient population data (194 left-breast and 103 right-breast cases). Model analytics confirmed that institutional statistics remained within acceptable quality benchmarks across all evaluated structures, ensuring that the DVH predictions were consistent with the clinical objectives (constraints).

Furthermore, the tool was instrumental in identifying and cleaning outliers within the training database. By analyzing regression coefficients and inspecting plans with significant geometric or dosimetric deviations, cases that did not represent the desired clinical standard were excluded. This refinement process improved the model's predictive accuracy and robustness, ensuring that the RapidPlan algorithm generates reliable optimization objectives tailored to the specific anatomical characteristics of the Guatemalan patient population.

A summary of plan-level compliance with ASTRO 2026 benchmarks is illustrated in Figures 3-4. The majority of plans across both lateralities fell within the recommended (green) zone for PTV coverage, heart mean dose, ipsilateral lung V18Gy, spinal cord D0.035cc, and contralateral breast D10%. The contralateral lung V4.3Gy metric showed the widest spread, with a subset of left-sided plans exceeding the primary goal threshold, as discussed above.

## Discussion

The implementation of KBP models at LNCC demonstrates that high-quality radiotherapy planning can be standardized in resource-constrained LMIC settings through inter-institutional collaboration. Preliminary results from this work were previously presented at the ASTRO 2023 Annual Meeting, where the standardization framework and early KBP outcomes were disseminated to the international radiation oncology community [13].

The dosimetric outcomes achieved in this study are consistent with findings from other institutions that have implemented RapidPlan KBP models for breast and chest wall VMAT. Fogliata et al. reported that KBP-based optimization outperformed manually optimized plans in 13% of dose-volume constraint assessments across three institutions, with notable improvements in cardiac dose [9]. Rago et al. validated 11 KBP models for whole-breast VMAT with locoregional nodal coverage, demonstrating reductions in heart maximum dose of approximately 2 Gy over clinical plans [10]. Phurailatpam et al. validated a RapidPlan model for post-mastectomy locoregional radiotherapy using a 40 Gy/15-fraction scheme--directly comparable to the present cohort--and reported dosimetrically superior or equivalent plans to those manually generated [14]. The present study extends these findings to a resource-constrained LMIC setting, demonstrating that comparable plan quality can be achieved through inter-institutional knowledge transfer. The collaboration with Washington University in St. Louis was not merely a technological transfer but a catalyst that shortened the institutional learning curve substantially by adapting validated models to the Guatemalan patient population. Complex VMAT treatments were standardized, ensuring that plan quality is governed by a robust institutional framework rather than individual planner experience [15,16].

A subset of plans had V95% values below the ASTRO 2026-recommended threshold. These cases correspond predominantly to the post-mastectomy chest wall subgroup, which represents approximately 70% of the present cohort, consistent with findings reported by Phurailatpam et al. for automated VMAT planning in similar anatomical settings. These suboptimal coverage values are predominantly associated with post-mastectomy chest wall cases in which the anatomical geometry presents a critical dosimetric challenge: the external surface of the patient lies in proximity to the underlying organs at risk, leaving insufficient tissue thickness between the skin edge and structures such as the heart and ipsilateral lung (Figure 1). In this anatomical context, achieving full PTV coverage to the prescribed dose would require delivering a higher dose to the adjacent OARs, creating an inherent trade-off between target coverage and normal tissue sparing. The KBP model, trained to respect OAR dose constraints, systematically prioritizes organ protection in geometrically unfavorable cases, leading to reduced PTV coverage. This behavior reflects a planning philosophy, widely endorsed in the literature for chest wall radiotherapy, in which dosimetric compromise at the target periphery is accepted when necessary to avoid exceeding critical organ tolerances. Importantly, the clinical significance of mild under coverage in post-mastectomy chest wall irradiation must be interpreted in the context of the overall treatment intent. For patients with no residual breast tissue, the therapeutic objective is locoregional disease control rather than dose homogeneity within a breast volume. Similar findings have been reported by other groups implementing automated planning for chest wall cases, confirming that this represents an inherent anatomical constraint rather than a model deficiency [14].

The use of RapidPlan enabled automated optimization, standardized planning objectives, and reduced inter-planner variability without sacrificing dosimetric quality. Comparing our metrics with those in ASTRO 2026 Table 3, we observe that KBP achieves consistent solutions even in challenging anatomical regions (such as the chest wall), where dose dispersion and ipsilateral lung protection are critical.

The integration of ESAPI extraction for automated data auditing enabled real-time epidemiological and dosimetric surveillance. Having our own statistics, validated against international guidelines, allows us to ensure that each of our patients receives treatment that meets the highest safety standards, minimizing the risk of long-term toxicities in organs such as the heart and spinal cord.

## Conclusions

Our findings confirm that the RapidPlan models, developed over a multi-year collaboration (2022-2024) and validated with 2025 clinical data, provide a reliable, standardized solution for breast radiotherapy in high-demand clinical environments such as LNCC. The successful alignment of these results with the ASTRO 2026 standards underscores the efficacy of knowledge-based planning in bridging the technological gap in LMICs. This approach demonstrates guideline-consistent dosimetric performance and supports the standardization of treatment planning at scale, establishing a sustainable framework for long-term quality assurance in resource-constrained environments. The dosimetric outcomes achieved are expected to contribute to reduced treatment-related morbidity, though prospective clinical validation remains warranted.
